# Predominant envelope variable loop 2-specific and gp120-specific antibody-dependent cellular cytotoxicity antibody responses in acutely SIV-infected African green monkeys

**DOI:** 10.1186/s12977-018-0406-5

**Published:** 2018-03-09

**Authors:** Quang N. Nguyen, David R. Martinez, Jonathon E. Himes, R. Whitney Edwards, Qifeng Han, Amit Kumar, Riley Mangan, Nathan I. Nicely, Guanhua Xie, Nathan Vandergrift, Xiaoying Shen, Justin Pollara, Sallie R. Permar

**Affiliations:** 1 Duke Human Vaccine Institute, Duke University School of Medicine, Durham, NC USA; 2 Department of Molecular Genetics and Microbiology, Duke University School of Medicine, Durham, NC USA; 3 Department of Surgery, Duke University School of Medicine, Durham, NC USA; 4 Department of Pediatrics, Duke University School of Medicine, Durham, NC USA; 5 Department of Immunology, Duke University School of Medicine, Durham, NC USA

**Keywords:** SIV, Linear peptide antibody responses, Natural SIV host, African green monkey, Rhesus monkey, Antibody response, ADCC, Envelope, gp120, gp41

## Abstract

**Background:**

The initial envelope (Env)-specific antibody response in acutely HIV-1-infected individuals and simian immunodeficiency virus (SIV)-infected rhesus monkeys (RMs) is dominated by non-neutralizing antibodies targeting Env gp41. In contrast, natural primate SIV hosts, such as African green monkeys (AGMs), develop a predominant Env gp120-specific antibody response to SIV infection. However, the fine-epitope specificity and function of SIV Env-specific plasma IgG, and their potential role on autologous virus co-evolution in SIV-infected AGMs and RMs remain unclear.

**Results:**

Unlike the dominant linear gp41-specific IgG responses in RMs, SIV-infected AGMs demonstrated a unique linear variable loop 2 (V2)-specific plasma IgG response that arose concurrently with high gp120-directed antibody-dependent cellular cytotoxicity (ADCC) activity, and SIVsab-infected cell binding responses during acute infection. Moreover, SIV variants isolated from SIV-infected AGMs exhibited high amino acid mutation frequencies within the Env V1V2 loop compared to those of RMs. Notably, the linear V2-specific IgG epitope in AGMs overlaps with an analogous region of the HIV V2 loop containing the K169 mutation epitope identified in breakthrough viruses from RV144 vaccinees.

**Conclusion:**

Vaccine-elicited Env V2-specific IgG responses have been proposed as an immune correlate of reduced risk in HIV-1/SIV acquisition in humans and RMs. Yet the pathways to elicit these potentially-protective V2-specific IgG responses remain unclear. In this study, we demonstrate that SIV-infected AGMs, which are the natural hosts of SIV, exhibited high plasma linear V2-specific IgG binding responses that arose concurrently with SIV Env gp120-directed ADCC-mediating, and SIV-infected cell plasma IgG binding responses during acute SIV infection, which were not present in acutely SIV-infected RMs. The linear V2-specific antibody response in AGMs targets an overlapping epitope of the proposed site of vaccine-induced immune pressure defined in the moderately protective RV144 HIV-1 vaccine trial. Identifying host factors that control the early elicitation of Env V2-specific IgG and ADCC antibody responses in these natural SIV hosts could inform vaccination strategies aimed at rapidly inducing potentially-protective HIV-1 Env-specific responses in humans.

**Electronic supplementary material:**

The online version of this article (10.1186/s12977-018-0406-5) contains supplementary material, which is available to authorized users.

## Background

The HIV-1 Env glycoprotein contains multiple vulnerable epitopes targeted by potent broad neutralizing antibodies (bNAbs) [1]. However, the elicitation of HIV gp120-specific bNAbs by current Env vaccination strategies is not yet feasible [2, 3]. Thus, HIV vaccine candidates currently in clinical testing focus on the elicitation of antibody specificities and functions identified as potential immune correlates of reduced infection risk in human and non-human primate vaccine efficacy studies. Immune analyses from the HVTN 505 phase IIb vaccine trial, which utilized an HIV Env gp140 protein boost immunogen and failed to show efficacy, demonstrated that the vaccine-elicited humoral responses primarily targeted HIV Env gp41 without identifiable antiviral functions [4]. Similarly, in the setting of HIV-1 infection, the initial antibody response against HIV Env is also dominated by Env gp41-specific IgG responses that are ineffective at controlling viremia [5, 6]. Interestingly, in the moderately-efficacious RV144 HIV-1 Env vaccine efficacy trial, Env-specific IgG responses targeting the variable loop 1 and 2 (V1V2) were found to be associated with reduced HIV acquisition risk [7]. The following sieve analysis of the breakthrough virus variants localized the site of vaccine-induced immune pressure to two amino acid residue positions within V2 loop [8–10]. Moreover, the V2 epitope that was associated with immune escape in RV144 vaccinees spans the region capable of engaging the gut-homing integrin receptor α4β7, which has been implicated in the trafficking of immune cells to the gut associated lymphoid tissue, and the enhancement of cell-to-cell HIV transmission [11–13]. Furthermore, the HIV Env V2-specific IgG responses in RV144 vaccinees mediated ADCC activity [14, 15]. Notably, V2-specific IgG responses were also associated with a reduced risk of SIVmac251 acquisition in RMs that received a similar vaccine regimen to that in the RV144 HIV-1 trial [16]. While these types of V2-specific IgG responses are a major clinical endpoint of HIV vaccination, our understanding of factors that control the elicitation of V2-specific IgG responses and ADCC function by existing vaccination strategies remains limited [3, 17].

Previous studies have investigated the proportion of SIV Env-specific IgG responses in the setting of infection in natural and non-natural SIV hosts. Antibody responses in acute SIV infection of RMs—a non-natural SIV host species and model of AIDS pathogenesis—predominantly target the SIV Env gp41 region [18–20]. Moreover, in SIV infected RMs, Env-specific IgG autologous virus neutralizing responses do not arise until approximately 1 year post-infection [18]. In contrast, AGMs—which are thought to have co-evolved with SIV for at least 30,000 years—sustain a non-pathogenic SIV infection, and do not exhibit a predominant gp41-specific IgG response over the course of infection [18, 19, 21]. SIV Env-specific IgG responses in chronically SIV-infected AGMs more frequently target gp120 epitopes compared to SIV-infected RMs [20]. While previous studies reported that B cell depletion in SIV-infected AGMs has no appreciable effect in disease progression outcome [22], it has also been shown that SIV-associated B cell dysfunction is associated with pathogenic SIV infection, and not in non-pathogenic SIV infection in natural SIV hosts [19, 23]. Moreover, we previously demonstrated that SIV Env gp120-specific IgG monoclonal antibodies (mAbs) isolated from chronically SIV-infected AGMs mediated robust virus capture activity, and ADCC—an antibody function that has been associated with delayed progression to AIDS in RMs [20, 24]. Moreover, SIV Env gp120-specific mAbs in chronically SIV-infected AGMs exhibited higher binding levels against SIV-infected CD4^+^ target cells compared to gp41-specific antibodies [20]. In this study, we further explore the kinetics of ADCC and SIV-infected cell binding responses, the fine epitope-specificity of the early Env-specific IgG responses in AGMs and RMs, and their potential role in autologous virus evolution at key vulnerable Env sites. A deeper understanding of the fine-specificity, kinetics, and antiviral function of gp120-specific IgG responses in AGMs may help guide future HIV vaccination strategies aimed at eliciting potentially-protective Env-specific IgG responses in humans.

## Results

### Kinetics of SIV Env-specific plasma IgG binding response in SIV-infected AGMs and RMs

**Fig. 1:**
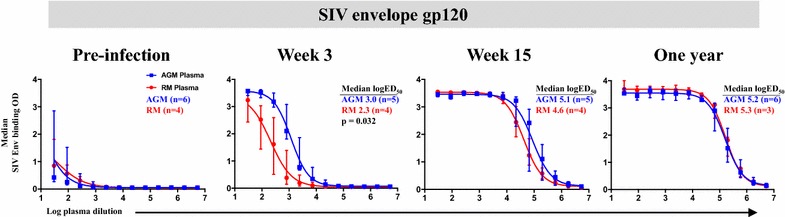
Early development of SIV Env gp120 IgG responses in SIV-infected AGMs compared to those in SIV-infected RMs. Recombinant SIVsab92018ivTF or SIVmac251 envelope (Env) gp120 were tested by ELISA using plasma samples from SIVsab92018ivTF-infected AGMs and SIVmac251-infected RMs throughout infection. Shown are the median OD binding curves with ranges for all monkeys against Env specificity and the corresponding median log half-maximal effective plasma dilution (logED_50_) at each time-point. Reported false discovery rate (FDR) *p* value by the Wilcoxon-Mann–Whitney test indicates significant difference in the logED_50_ values for each Env specificity between AGM and RM plasma binding at a given time-point

We previously showed that SIV-infected AGMs have a predominant gp120-specific antibody response compared to SIV-infected RMs [18]. In contrast, SIV-infected RMs have a more focused gp140-specific antibody response compared to SIV-infected AGMs [18]. We set out to further investigate the early acute kinetics of the gp120-specific IgG responses in AGMs and RMs by examining plasma IgG binding against the autologous SIVsab92018ivTF/SIVmac251 Env gp120 proteins in both species. Prior to SIV infection, AGM and RM plasma IgG exhibited relatively low binding against SIV Env proteins (Fig. 1). By 3 wpi, AGMs had higher magnitude SIV Env-specific IgG binding responses to the autologous SIV gp120 compared to RMs (logED_50_ median in AGMs vs. RMs: 3.0 vs. 2.3, FDR *p* = 0.032) (Fig. 1). By 15 weeks post-infection (wpi) and 1 year post-infection (1 ypi), there was no difference in plasma gp120-specific binding in SIVsab92018ivTF and SIVmac251-infected AGMs and RMs (Fig. 1).

### Linear SIV Env peptide-specific plasma IgG binding responses in SIV-infected AGMs and RMs

**Fig. 2:**
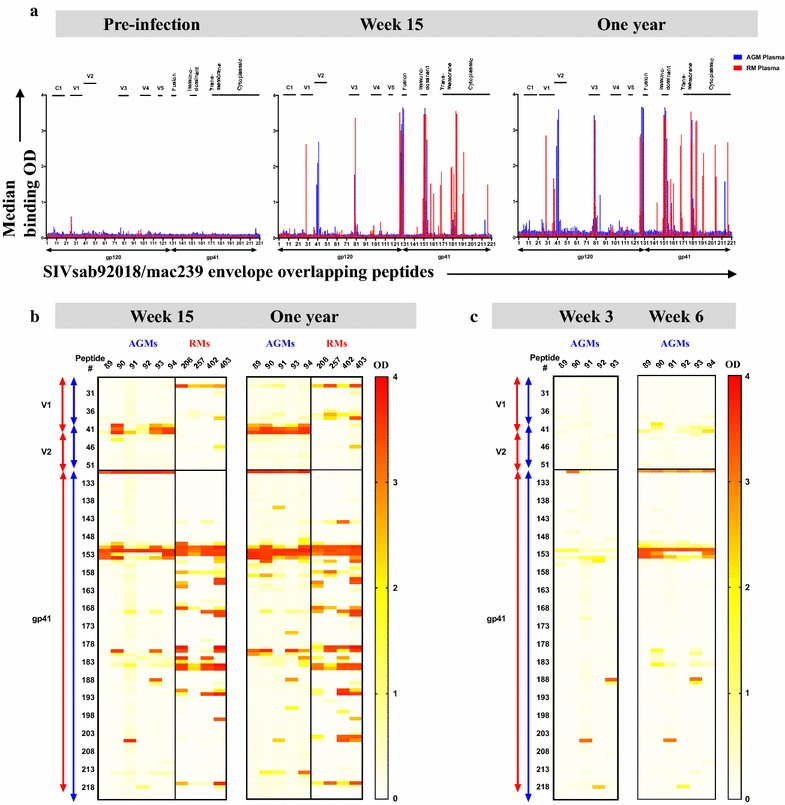
Distinct pattern of SIV Env linear peptide-specific plasma IgG responses in AGMs and RMs. Plasma samples from SIVsab92018ivTF-infected AGMs and SIVmac251-infected RMs during acute and chronic infection were tested against species-specific overlapping linear peptides spanning SIV envelope (Env) gp160 by ELISA. **a** Each peak represents the median plasma binding levels of all monkeys against each peptide across Env gp160. Each block represents the plasma binding level of **b** AGMs and RMs at week 15 and 1 year against each peptide within the V1, V2, and gp41 regions, or **c** AGMs at week 3 and week 6. Linear V2-specific plasma IgG response emerged at week 6 in AGMs. At week 15, AGM plasma showed a unique binding to a cluster of peptides in the V2 and V3 loops that increased in magnitude through chronic infection in addition to binding clusters nearby the gp120/gp41 fusion domain and immunodominant region of Env gp41

To map the fine-epitope specificity of SIV Env-specific IgG responses in SIV-infected AGMs and RMs, we measured plasma IgG responses against a linear overlapping peptide library spanning the entire species-specific SIVsab92018WT/SIVmac239 Env gp160 for each species. SIV Env linear peptide-specific plasma IgG binding responses were undetectable prior to infection (Fig. 2a). By 15 wpi, both species demonstrated strong plasma antibody responses against peptides analogous to the HIV gp120-gp41 fusion domain, gp41 immunodominant region, and the N-terminal region of gp41 cytoplasmic tail (Fig. 2a) [5]. Notably, by 15 wpi, RM plasma demonstrated high IgG binding against peptides of the variable loop 1 (V1) and variable loop 3 (V3) regions as well as binding to a large number of peptides within the gp41 subunit, including those of the membrane-proximal external region (MPER), which remained high binding responses at 1 ypi (Fig. 2a). In addition to an appreciable linear V3-specific IgG response by 15 wpi (Fig. 2a), 3 of 6 AGMs (AGMs 90, 93, 94) exhibited strong linear V2-specific IgG response that was markedly undetectable in all RMs (Fig. 2b). By 1 ypi, all AGMs had a high plasma IgG binding response against the linear V2 epitopes, yet this response remained undetectable in RMs (Fig. 2b). To more closely examine the kinetics of V2-specific IgG response in AGMs, we assessed AGM plasma IgG binding to the overlapping peptide library spanning SIVsab Env gp160 at earlier time-points during acute SIV infection. No appreciable plasma IgG binding to linear V2 peptides was detected at 3 wpi (Fig. 2c). Interestingly, in 3 of 6 SIV-infected AGMs (AGMs 90, 93, 94) plasma IgG binding responses against 3 overlapping linear V2 peptides appeared by 6 wpi (Fig. 2c).

### Autologous and heterologous V1V2- and linear V2-specific plasma IgG binding responses in SIV-infected AGMs and RMs

**Fig. 3:**
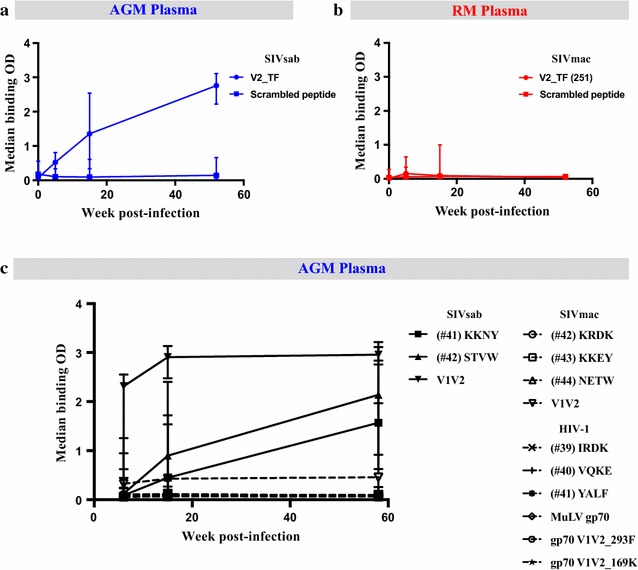
Autologous and heterologous V1V2- and linear V2-specific plasma IgG binding responses in SIV-infected AGMs. Autologous SIVsab92018ivTF and SIVmac251 linear V2 peptides were tested by ELISA using plasma samples from **a** SIVsab92018ivTF-infected AGMs (n = 5 for 15 wpi, and n = 6 for all other time-points), and **b** SIVmac251-infected RMs (n = 3 for 5 wpi, and n = 4 for all other time-points) throughout infection. **c** Heterologous plasma linear V2-specific IgG binding in 6 SIVsab92018ivTF-infected AGMs was assessed against SIVsab92018WT, and SIVmac239 and HIV-1 Consensus subtype B (ConB) overlapping linear V2 peptides with (#) indicate peptide number, and the last four amino acids from each peptide. Cross-reactivity of AGM V1V2-specific plasma IgG binding was also tested using SIVsab92018ivTF, and SIVmac251 and HIV-1 ConB V1V2 proteins by ELISA. Shown are the median OD binding curves with range for all monkeys against Env specificity at each time-point

Understanding the development of the predominant V2-specific IgG responses in AGMs is of interest, as it remains unclear how to elicit HIV V1V2-specific IgG responses that correlated with reduced HIV acquisition risk in the moderately protective RV144 vaccine trial [7–10]. To account for the few existing amino acid (AA) differences in the overlapping SIVsab92018WT and SIVmac239 V2 peptide libraries tested (peptides 40–42, and 42–44, respectively) compared to the AGM and RM challenge viruses, we measured AGM and RM plasma IgG binding against linear peptides that cover the same V2 region and directly match the AA sequence of the SIVsab92018ivTF (SFAMAGYRRDVKKNYSTVWYDQE) and SIVmac251 (KFNMTGLKRDKTKEYNETWYSTD) challenge viruses. SIV-infected AGMs exhibited high plasma IgG binding against the linear V2 SIVsab92018ivTF peptide at 15 wpi (median binding OD, 1.35 [range, 0.34–2.54]) and 1 ypi (median binding OD, 2.76 [range, 2.22–3.11]) (Fig. 3a), whereas RM plasma remained non-reactive against the linear V2 SIVmac251 peptide (median binding OD, 0.04 [range, 0.02–0.11] at 1 ypi) (Fig. 3b).

To examine the cross-reactivity of the predominant V2- and V1V2-specific IgG responses in SIV-infected AGMs, we assessed binding responses to both autologous and heterologous SIVmac and HIV-1 linear V2 peptides and V1V2 proteins. The V2-specific IgG response in AGMs was not cross-reactive with heterologous V2 linear SIV and HIV peptides or V1V2 proteins (Fig. 3c). However, SIV-infected AGM plasma demonstrated strong IgG binding to the autologous SIVsab Env V1V2 protein as early as 6 wpi (median binding OD, 2.3 [range, 0.6–2.6]) and this response increased in magnitude (median binding OD, 2.9 [range, 2.5–3.1]) by 15 wpi, and (median binding OD, 3.0 [range, 2.8–3.2]) by 1 ypi.

### SIV Env mutation frequency of autologous circulating viruses isolated from plasma of SIV-infected AGMs and RMs

**Fig. 4:**
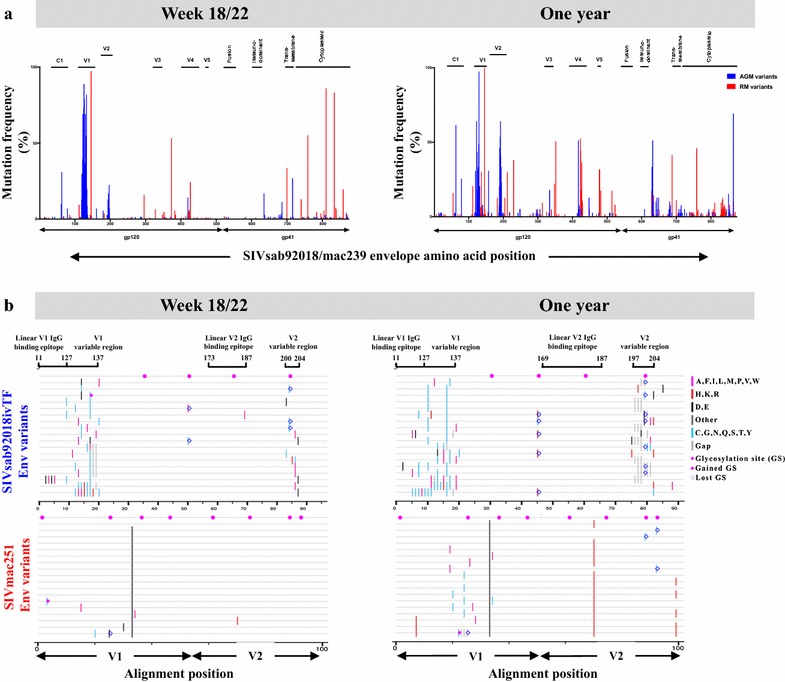
High mutation frequency in V1 and V2 loops of SIV Env variants in SIV-infected AGMs. **a** Mutation frequency in the protein sequence of plasma SIV Env variants isolated from SIVsab92018ivTF-infected AGMs and SIVmac251-infected RMs was quantified as the percent of Env variants showing a different amino acid compared to that of the challenge viral Env from the total number of variants. **b** Representative protein sequence alignments within V1V2 region of plasma SIV Env variants (depicted by each line) with mismatches highlighted relative to the respective wild-type challenge env (the top line). Hanging bars with SIVmac239 Env sequence number indicate relative positions of linear SIVmac V1 or SIVsab V2 IgG epitopes, and the two regions having high protein sequence variability in V1 and V2 loops. Purple circles indicate potential N-linked glycosylation sites (GS). Purple diamonds or blue empty diamonds indicate gained or lost potential N-linked GS in Env variants compared to corresponding challenge viral Env sequences, respectively

To explore the potential role of the predominant gp120- and V1V2-specific IgG binding responses in AGMs and gp41-specific IgG responses in RMs on autologous viral evolution, we examined the Env gp160 AA sequence variability of single genome Env variants isolated from plasma of SIVsab92018ivTF-infected AGMs at 22 wpi and 1 ypi, and SIVmac251-infected RMs at 18 wpi and 1 ypi. Plasma SIV Env variants from both species demonstrated appreciable mutation frequency in the region of gp120 V4 loop at 1 ypi (Fig. 4a). Yet, the Env variants isolated from SIV-infected RMs exhibited high variability in Env gp41 region, compared to Env variants from SIV-infected AGMs (Fig. 4a). By 1 ypi, Env variants in RMs also showed appreciable sequence variability in a region near the V3 and V5 loops (Fig. 4a). In contrast, plasma Env variants isolated from AGMs showed high sequence variability within C1 region that increased in frequency throughout infection compared to those of SIV-infected RMs (Fig. 4a). Moreover, plasma SIVsab Env variants in AGMs accumulated higher mutation frequencies within V1 (mutation frequency mean in AGM vs. RM variants, 5.4 vs. 0.22% at 22 or 18 wpi, and 6.7 vs. 3.5% at 1 ypi, respectively), and V2 loops (mutation frequency mean in AGM vs. RM variants, 1.8 vs. 0.086% at 22 or 18 wpi, and 5.9 vs. 3.2% at 1 ypi, respectively) compared to RM Env variants over the course of infection (Fig. 4b). Particularly, AGM plasma SIVsab Env variants exhibited high sequence variability at AA residue positions 127–137 within the V1 loop, and 200–204 within the V2 loop at 22 wpi and at 197–204 within the V2 loop at 1 ypi (SIVmac239 Env number sequence) (Fig. 4b). The high mutation frequency regions within SIVsab92018ivTF V2 are both near the epitope of the high magnitude linear V2-specific responses and within the vicinity of an analogous region of the HIV V2 loop where the K169 mutation hotspot was identified in breakthrough viruses isolated from HIV RV144 vaccinees (Fig. 4b) [9]. This SIVsab V2 loop site of high sequence variability is also near the analogous putative gut-homing receptor α4β7-binding motif identified in the HIV-1 Env (Fig. 4b) [11].

As N-linked glycans on Env can evolve in response to the autologous virus-specific IgG responses, we also assessed the N-linked glycosylation pattern in SIV plasma Env variants. AGM SIVsab Env plasma variants exhibited a relatively high loss of predicted N-linked GS motifs within the V2 variable region compared to RM SIVmac Env variants (percent of variants exhibiting a GS loss in AGMs vs. RMs: 35% at 22 wpi and 69% at 1 ypi vs. 0% at 18 wpi and 16% at 1 ypi, respectively) at equivalent AA positions (SIVmac239 Env position 202) (Fig. 4b). SIVsab Env variants also showed a 7 and 31% loss of predicted GS motifs (SIVmac239 Env position 167) at 22 wpi and 1 ypi, respectively, within the V1 loop. A loss in GS motifs within the corresponding V1 region was not observed in SIVmac Env variants (Fig. 4b). However, RM SIVmac Env variants isolated at 1 ypi had 30 and 16% loss of predicted GS motifs within the V5 loop that were not observed in AGM SIVsab Env variants from 1 ypi (SIVmac239 Env positions 476 and 479, respectively).

### ADCC kinetics of plasma IgG responses in SIV-infected AGMs and RMs

**Fig. 5:**
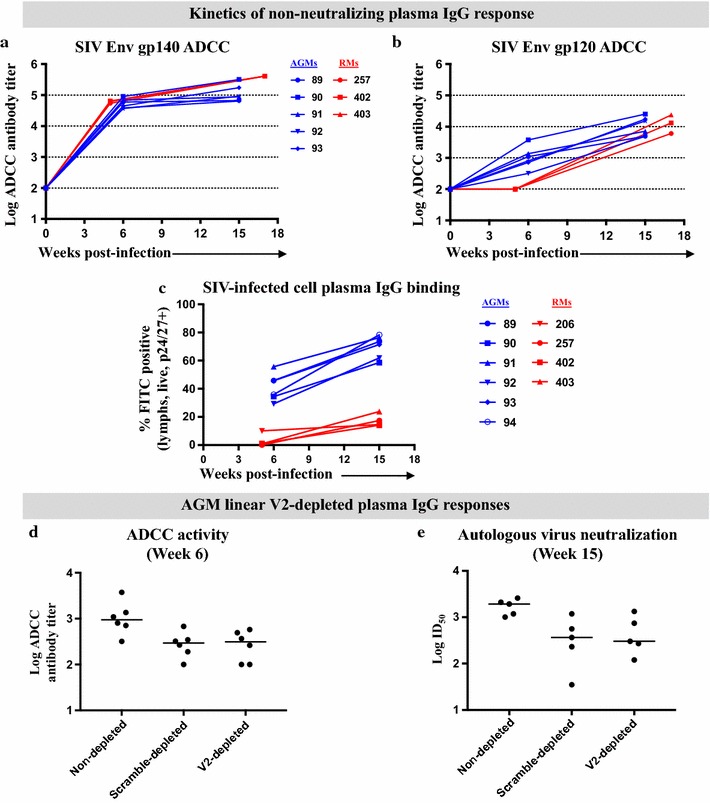
Early development of SIV Env gp120-directed plasma IgG ADCC and SIV-infected cell binding in AGMs. ADCC activity against the autologous challenge SIV envelope (Env) **a** gp140 and **b** gp120-coated cells of plasma IgG from SIVsab92018ivTF-infected AGMs and SIVmac251-infected RMs prior to infection, at week 6 (AGM) or 5 (RM), and 15 (AGM) or 17 (RM) was quantified as ADCC antibody titers. **c** Binding to SIV-infected cells was measured as % FITC positivity after week 0 background subtraction. **d** Plasma gp120-specific ADCC responses (median shown) after depletion with a linear V2 peptide or scrambled peptide in SIV-infected AGMs at week 6. **e** Neutralizing responses (median shown) in SIVsab92018ivTF-infected AGMs against the autologous challenge virus at week 15 after depletion with a linear V2 peptide and scrambled peptide

ADCC-mediating IgG responses and IgG targeting linear V2 epitopes were associated with a decreased risk of HIV-1 infection in the RV144 vaccine trial [25]. To explore the ADCC function of Env gp120-predominant antibody responses and their relationship to the early development of linear V2-specific IgG responses in AGMs, we evaluated the kinetics of plasma Env-specific ADCC responses in AGMs compared to those of RMs. ADCC activity was not detected in AGMs or RMs prior to SIV infection (Fig. 5a, b). As early as 6 wpi in AGMs and 5 wpi in RMs, high ADCC activity was detected against SIV Env gp140-coated cells in both species (ADCC titer as reciprocal plasma dilution median in AGMs vs. RMs: 52,225 [range, 36,931–90,286] vs. 61,826 [range, 54,503–64,725], raw *p* = 0.71, FDR *p* = 0.95) (Fig. 5a). Gp140-specific IgG-mediated ADCC activity increased in both species by 15 wpi in AGMs and 17 wpi in RMs (ADCC titer median in AGMs vs. RMs: 89,851 [range, 64,474–317,680] vs. 409,600 [range, 409,600–409,600], raw *p* = 0.024, FDR *p* = 0.063) (Fig. 5a). Interestingly, SIV-infected AGMs demonstrated high magnitude ADCC activity against SIV Env gp120-coated cells at 6 wpi, and this response was remarkably undetectable in acutely SIV-infected RMs (ADCC titer median in AGMs: 949.3 [range, 319–3746], raw *p* = 0.024, FDR *p* = 0.063) (Fig. 5b). Importantly, the more rapid development of gp120-specific ADCC-mediating response in AGMs arose concurrently with the gp120 and V2 loop-specific antibody responses in AGMs, which are distinct from that in SIV-infected RMs. Yet, both AGMs and RMs had comparable ADCC-mediating gp120-specific IgG responses by 15 or 17 wpi (ADCC titer median in AGMs vs. RMs: 11,169 [range, 4867–25,125] vs. 13,120 [range, 6036–23,722], raw *p* = 0.90, FDR *p* = 1) (Fig. 5b).

### Plasma IgG binding to SIV-infected CD4^+^ T cells

To examine the infected cell binding activity of the early acute SIV Env-specific IgG response in AGMs and RMs, we measured the binding of Env-specific IgG to CD4^+^ T cells infected with SIVsab92018ivTF and SIVmac251 infectious molecular clones (IMC). For all experiments, we obtained high levels of infection with > 55% (range, 56–82% p24/p27+) of cells being infected for both viruses. Env-specific IgG plasma responses in AGMs recognized SIV-infected CD4^+^ target cells more effectively and earlier in acute time points compared to RM plasma (median % FITC positivity in AGMs vs. RMs, 40.8% [range, 29.3–55.6%] vs. 1.0% [range, 0.1–1.1%] at week 6 or 5, raw *p* = 0.0095, FDR *p* = 0.057; 72.5% [range, 58.6–78.3%] vs. 23.2% [range, 17.3–24.6%] at week 15 or 17, raw *p* = 0.0095, FDR *p* = 0.057, respectively) (Fig. 5c).

### Limited contribution of linear V2 epitopes to early ADCC and autologous SIV neutralizing responses in SIV-infected AGMs

Given the early development of ADCC and neutralizing activity that arose concurrently with the linear V2-specific plasma IgG binding response in AGMs during acute SIV infection, we sought to define potential contribution of the linear V2-specific IgG response to early gp120-directed functional responses that are unique to AGMs. With a SIVsab linear V2 peptide-coated IgG depletion column of AGM plasma, we achieved an enriched 76 ± 4.4% (mean and SD) depletion of linear V2-specific antibodies compared to 53 ± 8.4% (mean and SD) depletion with a scrambled peptide-coated column. We then compared the gp120-specific ADCC and autologous neutralizing function of AGM V2 and scrambled peptide-depleted plasma at 6 and 15 wpi, respectively [18]. Despite a reduction in ADCC activity compared to that of non-depleted plasma (median ADCC titers for non-depleted vs. V2-depleted response, 949.3 [range, 318.8–3746] vs. 314.4 [range, 100–576.3], raw *p* = 0.031, FDR *p* = 0.063; non-depleted vs. scrambled peptide-depleted response, 294.6 [range, 100–679.5], raw *p* = 0.031, FDR *p* = 0.063), we observed similar ADCC antibody titers in linear V2 peptide and scrambled peptide-depleted plasma at 6 wpi (raw *p* = 1, FDR *p* = 1) (Fig. 5d). Similarly, we observed an equivalent decrease in plasma SIVsab neutralizing activity following the linear V2 and scrambled peptide-depletion of AGM plasma at 15 wpi (median ID_50_ for non-depleted vs. V2-depleted activity, 1933 [range, 1007–2592] vs. 303 [range, 120–1340], raw *p* = 0.13, FDR *p* = 0.19; non-depleted vs. scrambled peptide-depleted activity, 367 [range, 35–1184], raw *p* = 0.063, FDR *p* = 0.11; scrambled peptide-depleted vs. V2-depleted activity, raw *p* = 1, FDR *p* = 1) (Fig. 5e). AGM and RM plasma IgG binding to scrambled peptide was negligible (median binding OD, 0.21 [range, 0.15–0.25], and 0.08 [range, 0.04–0.12], respectively).

## Discussion

Vaccine-elicited V2-specific IgG responses that mediate ADCC activity have been proposed as an immune correlate of reduced HIV-1 infection risk in the moderately protective RV144 vaccine efficacy trial [3, 17, 26]. However, factors controlling the elicitation of V2-specific IgG responses capable of mediating ADCC are not well established. We previously reported that natural SIV hosts, AGMs, have a SIV Env gp120-biased antibody response to SIV infection, and rapidly develop autologous neutralizing antibodies, which are unique from both SIV-infected RMs and HIV-infected human non-natural hosts [18–20]. In this study, we identified that the distinct plasma linear V2-specific IgG responses arose at 6 wpi in 50% of all AGMs, which were markedly undetectable in all RMs. Moreover, acutely SIV-infected AGMs mediated a more rapid development of gp120-directed ADCC activity, and SIV-infected cell binding response compared to SIV-infected RMs. In addition, single genome Env variant analyses demonstrated an increase in AA mutation frequency, and a high loss of predicted N-linked GS within the V1 and V2 loops of SIVsab Env variants over time. Interestingly, the target of linear V2-specific plasma IgG binding responses in AGMs localized to an equivalent HIV V2 loop epitope near residues that were associated with immune escape from the potentially-protective V2-specific IgG responses in RV144 vaccinees [8, 9]. This SIVsab linear V2 epitope is also in close vicinity of the putative binding motif of the gut-homing receptor α4β7 in HIV-1 Env, underlining the potential importance of the V2 loop region for virus infectivity and escape from humoral responses in the natural SIV hosts.

In this study, we found that SIV-infected AGMs exhibited a predominant SIV Env gp120-directed response very early during acute SIV infection compared to the RM counterparts. To further examine the gp120/gp41 antibody differential antibody responses in these species, we measured their SIV Env linear peptide-specific IgG binding responses against overlapping SIVsab92018WT and SIVmac239 peptide libraries spanning Env gp160, respectively. Interestingly, we observed higher binding responses against linear V2 peptides in AGMs but not in RMs. Given that a few AA differences occur in these overlapping peptide libraries compared to the SIVsab92018ivTF and SIVmac251 challenge viruses, we also tested AGM and RM plasma IgG binding against linear V2 peptides matching the challenge viruses. AGMs showed higher magnitude linear V2-specific IgG binding against the linear V2 SIVsab92018ivTF peptide compared to RM plasma binding to linear V2 SIVmac251 peptide, suggesting that the observed binding differences against linear V2 peptides in AGMs and RMs are not due differences in the AA sequences between the SIV Env overlapping peptide library and the SIV challenge viruses. While the mechanism of the predominant, early plasma linear V2-specific IgG response in AGMs remains unclear, it is possible that AGMs mediate a higher frequency of SIV linear-epitope specific responses given their lower frequency of SIV immune-complex trapping by follicular dendritic cells in germinal centers [27, 28].

We observed that V1 and V2 loops of SIVsab variants had higher mutation frequencies compared to SIVmac variants isolated from RMs. This high Env mutation frequency in the V1V2 region has also been reported in SIVsab isolates from naturally SIV-infected AGMs [29, 30]. Therefore, it is plausible that the early development of predominant gp120-specific and high magnitude V2-specific IgG responses in AGMs may influence the unique Env mutation pattern observed within the V1V2 loop of autologous SIVsab circulating viruses [25, 31, 32]. It is also worth noting that the hypervariable loops of HIV-1 have long been suspected to influence conformational state changes in Env [33]. In addition, mutations within V1 or V2 loop could influence the conformation of nearby epitopes via glycan shielding [31, 32, 34, 35].

Comparisons in SIV and HIV Env-specific antibody responses are admittedly limited by a lack of SIV Env trimer crystal structures [32, 36–38]. The structure of a core SIV gp120 monomer has been determined and differed significantly in the orientation of its inner and outer domains compared to HIV gp120 monomers, suggesting a differing functional SIV Env trimer arrangement ([39]). Nevertheless, SIV V1V2 has been recognized to correspond to HIV-1 V1V2, and the SIV and HIV V2 loops share certain structural motifs, specifically the [ED][VLI] motif and a lysine-rich motif that appears as K[KV]QK in HIV and KK (alternately, K[KT]K) in SIV [29, 36, 37, 40]. In fact, an RV144 vaccine regimen utilizing SIV gp120 as the immunogen elicited V2 loop-specific IgG responses that were associated with protection against SIVmac251 in RMs [16]. Furthermore, V2-specific antibody responses were inversely correlated with peak and set-point viral loads in SIV-infected RMs [41]. Thus, the V2 loop in HIV and SIV may share immunogenic and functional characteristics that are capable of eliciting potentially-protective IgG responses in non-human primates.

Vaccine-elicited V2-specific IgG responses from the RV144 vaccine efficacy trial may have mediated protection through an ADCC mechanism [7, 9, 15]. In addition to the previously reported early kinetics of autologous virus neutralizing responses in AGMs [18], we observed an early SIV Env gp120-specific ADCC response in AGMs compared to RMs. Moreover, AGM plasma IgG showed higher SIV-infected cell binding activity compared to RM plasma, suggesting that AGM SIV Env-specific IgG responses may better recognize epitopes exposed on infected cells. Similar functional immune profiles using AGM sera have been observed in previous studies of naturally SIV-infected AGMs [42, 43]. These results suggest that AGM SIV Env-reactive plasma IgG can mediate robust non-neutralizing antiviral functions and increased infected cell binding activity during early acute SIV infection compared to RM plasma antibodies. Intriguingly, we observed similar ADCC activity in V2 and scrambled peptide-depleted AGM plasma, suggesting that other sites within SIVsab gp120 may mediate the early acute ADCC responses. However, it should be noted that there was non-specific IgG depletion in the scrambled peptide depletion experiments [44]. Furthermore, vaccine-elicited antibody responses in the RV144 efficacy trial were mapped to both conformational and linear V2 epitopes, suggesting that V2-specific IgG responses with anti-viral activity may not only target linear V2 epitopes [9, 14]. Interestingly, it has been shown that conformational epitope recognition of SIV Env-specific antibody responses differs greatly between nonpathogenic and pathogenic SIV infections in natural SIV hosts [23]. Thus, ADCC-mediating IgG responses in humans and AGMs may target both linear V2 and conformational gp120 epitopes within HIV and SIV Env.

## Conclusion

We have demonstrated that acutely SIV-infected AGMs develop high magnitude plasma linear V2-specific IgG responses that arise concurrently with ADCC-mediating and SIV-infected cellular binding responses targeting gp120 epitopes. Yet, these early plasma linear V2-specific and gp120-specific IgG responses with ADCC activity were undetectable in RMs. Moreover, the antibody responses targeting either the linear V2 or other non-linear gp120 variable loop epitopes that are generated more quickly in AGMs than those in RMs could contribute to the observed faster accumulation of diversity of autologous circulating viruses within the V1V2 loop in SIV-infected AGMs. Our findings support the potential use of AGMs as an alternative non-human primate model for HIV-1 vaccine development, particularly for the evaluation of immunogens that target high magnitude neutralizing and non-neutralizing gp120-specific IgG responses. A greater understanding of host immune factors that lead to the unique functional gp120-specific IgG responses in these natural SIV hosts may provide valuable insights for future HIV vaccine immunogen design.

## Methods

### Nonhuman primates and sample collection

Six female AGMs (*Chlorocebus sabaeus*) and four female Indian RMs (*Macaca mulatta*) between four and 11 years of age were intravenously inoculated with, infectious molecular clones of SIVsab92018ivTF, or SIVmac251.30 virus isolate, respectively [19, 20, 45]. All four RMs maintained high-level viremia compared to those of AGMs over the course of infection [18]. Blood was collected in EDTA tubes at 0, 2, 3, 5, 6, 15, 17, and 18 (RMs) or 22 (AGMs) weeks post-infection (wpi), and again at 1 year post-infection (ypi) in both species, and plasma was then isolated. Animals were housed and maintained according to the *Guide for the Care and Use of Laboratory Animals* [46].

### HIV/SIV Env protein and peptides

To assess the fine-specificity of SIV Env-specific plasma IgG binding responses in AGMs and RMs, we tested plasma samples for IgG binding to recombinant SIVsab92018ivTF Env gp120, [47] SIVmac251 Env gp120, [47] HIV-1 murine leukemia virus (MuLV) gp70_His6/Mon (293F), gp70 V1V2 CaseA subtype B (293F), gp70 V1V2 CaseA2 subtype B (169 K), and [48] SIVsab92018ivTF or SIVmac251 V1V2 proteins [18]. To examine Env linear peptide IgG binding responses, we used the SIVagm Sabaeus 92018 (Cat #10451), SIVmac239 (Cat #6883, AAA47637.1), and HIV-1 ConB (Cat #9480) Env peptide sets obtained through the NIH NIAID AIDS Reagent Program, Division of AIDS in addition to SIVsab92018ivTF V2 peptide (SFAMAGYRRDVKKNYSTVWYDQE) and SIVmac251 V2 peptide (KFNMTGLKRDKTKEYNETWYSTD) (CPC Scientific, Sunnyvale, CA). SIVmac239 Env peptide regions were annotated based on the HIV-2/SIV protein annotations from HIV Sequence Compendium 2016 [49]. To annotate the regions for SIVsab92018 Env overlapping peptide, its sequence was aligned with SIVmac239 (M33262) using the HIVAlign tool (https://www.hiv.lanl.gov/content/sequence/VIRALIGN/viralign.html). An additional table file details the information of both SIVsab92018 and SIVmac239 Env overlapping peptides for each Env region (see Additional file 1).

### HIV/SIV Env-specific IgG binding enzyme-linked immunosorbent assays (ELISAs)

To assess SIV Env-specific plasma IgG binding kinetics, plasma samples from SIVsab92018ivTF-infected AGMs and SIVmac251-infected RMs were tested for IgG binding to recombinant SIVsab92018ivTF or SIVmac251 Env gp120 proteins at the following time-points: 0, 3, 15 wpi, and 1 ypi according to sample availability. To measure linear SIV Env peptide-specific plasma IgG binding responses, plasma samples from SIV-infected AGMs and RMs from 0, 3, 6, 15 wpi, and 1 ypi were tested for IgG binding to SIVsab92018WT and SIVmac239 overlapping linear peptide libraries, respectively. To confirm similar IgG responses to the autologous linear V2 peptides, plasma samples from SIV-infected AGMs and RMs at 5, 15 wpi, and 1 ypi were tested against SIVsab92018ivTF and SIVmac251 linear V2 peptides, respectively. To examine the magnitude and cross-reactivity of the heterologous linear V2 and V1V2 protein-specific plasma IgG binding, plasma samples from SIV-infected AGMs at 6, 15 wpi, and 1 ypi were serially diluted from a 1:100 starting dilution and tested against SIVmac239 and HIV-1 Consensus Subtype B (ConB) overlapping linear V2 peptides, and SIVsab92018ivTF, SIVmac251, and HIV-1 V1V2 proteins. The SIV Env/peptide-specific IgG binding ELISAs were carried out with Env proteins or peptides diluted to 3 μg/mL in 0.1 M sodium bicarbonate, and coated on plates for 1 h. Plasma samples were diluted at 1:100. Plates were washed and blocked with SuperBlock (4% whey protein, 15% goat serum, and 0.5% Tween 20 diluted in 1X phosphate-buffered saline (PBS)) at room temperature for 1 h. Plasma from chronically SIV-infected AGMs and RMs were used as positive controls. Influenza hemagglutinin-specific mAb CH65 [50] and an anti-respiratory syncytial virus antibody, Pavilizumab (Medimmune, Inc, Quakertown, PA), were used as negative controls. Scrambled peptides and species-specific SIV Env gp140 proteins were used as additional negative and positive controls, respectively. Horseradish peroxidase (HRP)-conjugated polyclonal goat anti-monkey IgG (gamma chain) antibody (Rockland Immunochemicals, Gilbertsville, PA) was added and incubated for 1 h at 1:10,000 dilution. Plates were washed and developed with SureBlue reserve TMB substrate for 5 min, and the reaction was stopped by adding an equal volume of TMB stop solution (KPL, Gaithersburg, MD). Optical densities (ODs) were measured at 450 nm using a Spectramax Plus spectrophotometer (Molecular Devices, Sunnyvale, CA). A line of best fit calculated by a 4-parameter logistic curve was used to interpolate the log half-maximal effective plasma dilution (log ED_50_) using GraphPad Prism 7 (Graph Pad, La Jolla, CA).

### Peptide depletion and SIV neutralization assays

To deplete linear V2-specific IgG responses, CNBr-activated Sepharose 4B columns (GE Healthcare, Pittsburgh, PA) were coated with 2 mg of SIVsab92018WT V2 peptide (SFAMAGYRRDVKKNYSTVWDDQE) (CPC Scientific, Sunnyvale, CA), and a scrambled peptide of similar length according to manufacturer’s guidelines. AGM plasma samples were run through the columns, and the flow-through was run for a total of 10 times. Depletion efficiency of linear V2 peptide-specific antibodies was quantified as the percent depletion, which equals 1- ((OD of peptide-depleted V2 binding/OD of non-depleted V2 binding) * 100). Non-depleted, linear V2 peptide-depleted, and scrambled peptide-depleted plasma samples from SIVsab92018ivTF-infected AGMs at 15 wpi were tested against SIVsab92018ivTF infectious molecular clone (IMC) in the TZM-bl cell neutralization assay [18]. In brief, samples were serially diluted in duplicate, and incubated with virus (50,000 relative light unit [RLU] equivalents) for 1 h at 37 °C. Cells were added at a density of 10,000 cells per well, and incubated for 48 h at 37 °C + 5% CO_2_. A reduction in luciferase activity was quantified by the Brightglow Luciferase detection system (Promega, Madison, WI). The sample dilution at which a 50% RLU reduction was observed was defined as the inhibitory dilution (ID_50_).

### ADCC assay

We used the GranToxiLux (GTL) ADCC assay to measure the ADCC activity of whole plasma samples, linear V2 peptide-depleted plasma IgG, and/or scrambled peptide-depleted plasma IgG isolated from SIVsab92018ivTF-infected AGMs and SIVmac251-infected RMs at 0, 6 (AGMs) or 5 (RMs), and 15 (AGMs) or 17 (RMs) wpi as described previously [51]. The CEM.NKR_CCR5_ target cells [52] were coated with recombinant SIVsab92018ivTF/SIVmac251 gp120, or avi-tagged gp140 Env representing the same virus isolates. Cryopreserved human peripheral blood mononuclear cells from an HIV-seronegative donor with the 158F/F polymorphic variant of Fcγ receptor 3A served as effector cells [53]. Plasma samples were tested using 4-fold serial dilutions starting at 1:100 dilution. Data were analyzed using FlowJo 9.8.2 (Tree Star Inc., Ashland, OR). The % Granzyme B (GzB) activity was defined as the percentage of cells positive for proteolytically active GzB out of the total viable target cell population. Final results were calculated after subtracting the background % GzB activity observed in wells containing effector and target cells in the absence of plasma or IgG samples. Plasma ADCC antibody titers were determined by interpolating the plasma dilutions that intersect the positive cut-off (8% GzB activity) using Graph Pad Prism 7 (Graph Pad, La Jolla CA).

### Plasma IgG binding to Env on the surface of SIV-infected CD4^+^ cells

Indirect surface staining was used to evaluate the ability of SIV Env-specific plasma IgG from AGMs and RMs to bind the surface of CEM.NKR_CCR5_ CD4^+^ T cells [54] infected with SIVsab92018ivTF or SIVmac251. Infections with replication competent infectious molecular clone virus (IMC) were performed using DEAE-Dextran as described previously [51, 55]. At 48 h postinfection, the infected CEM.NKR_CCR5_ cells were incubated with a 1:100 dilution of plasma for 2 h at 37 °C and then stained with a vital dye (Live/Dead Fixable Aqua Dead Cell Stain; Invitrogen) to exclude nonviable cells from subsequent analyses. Primary Ab binding was detected by secondary labeling with fluorescein isothiocyanate (FITC)-conjugated goat anti-rhesus IgG (SouthernBiotech Inc., Birmingham, AL), and SIV-infected cells were identified by staining for intracellular expression of p24/27 (KC57-RD1; Beckman Coulter) using standard methods. Binding was quantified as % of live, p24/27 positive, FITC positive cells after subtracting the background binding of week 0 samples. Mock-infected cells were used to establish negative gates.

### Single genome amplification (SGA) and sequencing of plasma SIV env variants

SIV env variants from SIVsab92018ivTF-infected AGM plasma samples at 22 and 52 wpi (n = 4, 71 variants, and n = 3, 39 variants, respectively), [56] and SIVmac251-infected RM plasma samples at 18 and 50 wpi (n = 4, 107 variants; and n = 3, 67 variants, respectively) were amplified and sequenced using limiting dilution PCR [56]. For this study, SGAs isolated from AGMs and RMs were aligned against sequences of the corresponding wild-type SIVsab92018 and SIVmac251 using Seaview [57]. For SIVsab92018WT Env sequence (871 amino acid (AA)), the regions include constant region 1 (C1) (54 AA), V1 (42 AA), V2 (45 AA), and gp41 (345 AA). For SIVmac251 Env sequence (876 AA), the regions include C1 (49 AA), V1 (58 AA), V2 (43 AA), and gp41 (348 AA). Mutation frequency of Env variants at each time-point was calculated as the percent of variants with different AA compared to respective SIVsab/mac virus Env sequence at each residue within a defined Env region. Mutations in Env variants were graphically represented in relationship to their respective challenge Env using Highlighter tool (https://www.hiv.lanl.gov/content/sequence/HIGHLIGHT/highlighter_top.html). Analysis of N-linked glycosylation sites (GS) (Nx[ST] pattern) of plasma Env variants were performed using N-GlycoSite tool (https://www.hiv.lanl.gov/content/sequence/GLYCOSITE/glycosite.html).

### Statistical analysis

Wilcoxon-Mann–Whitney was used to compare the Env protein-specific plasma IgG binding logEC_50_ values between AGMs and RMs (Fig. 1). The exact Wilcoxon Rank Sum test was used to compare the gp140 and gp120 ADCC titers in AGMs and RMs (Fig. 5a, b), and the % infected cells in AGMs and RMs (Fig. 5c). The Wilcoxon Signed-Rank test to compare the non-depleted and V2/scrambled peptide depleted ADCC activity (Fig. 5d), and neutralizing activity (Fig. 5e). All analyses were adjusted for multiple comparisons by false discovery rate (FDR) *p* value correction.

## Additional file

**Additional file 1: Table S1.** SIVsab92018WT and SIVmac239 overlapping peptide envelope regions. SIVsab92018WT and SIVmac239 Env overlapping peptides were categorized into C1, V1-V5, gp120 other, fusion domain, gp41 immunodominant region, transmembrane region, cytoplasmic tail, and gp41 other.
