# Supplementary material for: Predominant envelope variable loop 2-specific and gp120-specific antibody-dependent cellular cytotoxicity antibody responses in acutely SIV-infected African green monkeys
Source: Retrovirology. 2018 Mar 9;15:24. doi: 10.1186/s12977-018-0406-5 (PMC5845189; doi:10.1186/s12977-018-0406-5)
Supplement: Supplementary file 1 — Additional file 1: Table S1. SIVsab92018WT and SIVmac239 overlapping peptide envelope regions. SIVsab92018WT and SIVmac239 Env overlapping peptides were categorized into C1, V1-V5, gp120 other, fusion domain, gp41 immunodominant region, transmembrane region, cytoplasmic tail, and gp41 other. [file 12977_2018_406_MOESM1_ESM.docx]

**Additional file 1**

| **Table S1** **SIVsab92018WT and SIVmac239 overlapping peptide envelope regions** | | | | |
| --- | --- | --- | --- | --- |
|  | **SIVsab92018WT** | | **SIVmac239** | |
| **Envelope region** | **Number of peptides (Peptide ID)** | **Peptide sequence** | **Number of peptides (Peptide ID)** | **Peptide sequence** |
| C1 | 15 (6-20) | IPVWKNSSVQAFCKTPNTNLWASTNCIPDDEPEGTIAEVPIPNITEKFDAWKNRN | 15 (6-20) | VPAWRNATIPLFCATKNRDTWGTTQCLPDNGDYSEVALNVTESFDAWNN |
| V1 | 13 (27-39) | CYRLEGGAATTKTPSTTTARPEVVSVGYNDSVIEKEMEKEQAMN | 15 (27-41) | CNKSETDRWGLTKSITTTASTTSTTASAKVDMVNETSSCIAQDNCTGLEQEQMIS |
| V2 | 12 (40-51) | SFAMAGYRRDVKKNYSTVWDDQEVVCEEGREKSNATNTVGCYMIHC | 11 (42-52) | KFNMTGLKRDKKKEYNETWYSADLVCEQGNNTGNESRCYMNHC |
| V3 | 11 (75-85) | CRRPGNKTVLPVTIMAGLVFHSQKYNTRLKQAWC | 11 (76-86) | CRRPGNKTVLPVTIMSGLVFHSQPINDRPKQAWC |
| V4 | 12 (98-109) | CKMDWFLNYLNNQSVDPDHNPCNKTGDKKCWQRTYVPC | 10 (99-108) | CKMNWFLNWVEDRNTANQKPKEQHKRNYVPC |
| V5 | 4 (117-120) | FDSNSR | 4 (116-119) | WIDGNQ |
| Gp120 other | 77 |  | 78 |  |
| Fusion domain | 6 (130-135) | VPFVLGFLGFLGAAG | 6 (130-135) | GVFVLGFLGFLATAG |
| Gp41 immuno-dominant region | 7 (149-155) | EDQARLNIWGCAFRQVCHTT | 7 (149-155) | KDQAQLNAWGCAFRQVCHTT |
| Trans-membrane givregion | 7 (172-178) | AIMVIAGIIIARILFVIISL | 7 (172-178) | GVYIVVGVILLRIVIYIVQM |
| Cytoplasmic tail | 43 (179-221) |  | 40 (179-218) |  |
| Gp41 other | 29 |  | 29 |  |
|  | Total = 221 |  | Total =218 |  |
